# Linking bone marrow fat with decreased bone mineral density among Indian patients with osteoporotic fracture

**DOI:** 10.6026/973206300200049

**Published:** 2024-01-31

**Authors:** Modagan Paranthaman, K.S.V. Angu Bala Ganesh, Santhi Silambanan

**Affiliations:** 1 Department of Biochemistry, Dhanalakshmi Srinivasan Medical College and Hospital, Affiliated to The Tamilnadu Dr MGR Medical University, Perambalur 621 113, Tamil Nadu, India; 2 Department of Anatomy, Gujarat Adani Institute of Medical Science, Bhuj, Gujarat 370001, India; 3 Department of Biochemistry, Sri Ramachandra Medical College, Sri Ramachandra Institute of Higher Education and Research, Porur, Chennai 600 116, Tamil Nadu, India

**Keywords:** Osteoporosis, bone marrow fat, bone mineral density, bone fracture

## Abstract

Osteoporosis is a systemic skeletal disorder with low-bone mass causing micro-architectural deterioration and an increase in bone fragility and susceptibility to fractures. According to a worldwide report by IOF, 1 in 3 females and 1 in 5 males will experience fractures due to the osteoporotic changes in their bones. Fractures may be the first clinical manifestation of the disease. They have been causes for morbidity and mortality imposing economic burden to osteoporosis. Bone marrow fat is a negative regulator of bone-turnover and a key integrator of bone and energy metabolism. Hence we assess the bone marrow fat and BMD in patients with osteoporotic bone fractures. This cross-sectional study was conducted in 30 patients from the department of orthopaedic surgery. Biopsy samples were received from excised bone during surgery. Biochemical parameters and bone marrow fat were quantified by established methods. A negative correlation between BMD versus serum adiponectin, FGF21 and similar observation with BMD versus bone marrow fat is seen. Therefore, increased bone-marrow fat and adiponectin, FGF21 levels and decreased BMD in osteoporosis. This observation might be useful for prevention, management and therapeutic potential of osteoporosis. Based on our study findings, understand the bone-fat relationship to implications with low BMD in patients with osteoporosis.

## Background:

Bone marrow is a gelatinous tissue that fills the medullary cavities of the bones, which occupies approximately 85% of the bone cavity, and the rest of the portion filled with trabecular bone [1]. It is rich in vasculature and cellular components within the bone that is responsible for the production and delivery of blood cells. The marrow consists of red bone marrow (also known as myeloid tissue) and yellow bone marrow (fatty tissue). At birth bone marrow remains "red". As the age advances, the red marrow is gradually replaced by yellow fat tissue [2]. The differentiation of adipocytes or osteoblasts is regulated by a complex mechanism involving many growth and transcription factors. Normal aging process increases the process of adipogenesis due to physiological decrease in growth factors release as well as oxygen tension and blood supply in bone marrow. This results in an increase of adipose tissue in the bone cavity with advancing age [3].

Ciril Rozman *et al*. demonstrate that the physiologic phenomenon is maintained during different physiopathologic situations such as bone marrow aplasia, hyperplasia, and dysplasia. An important degree of variation in size and number can be observed in these abnormal conditions. As expected, the capacity of adipocytes to expand and increase their size is not unlimited. In hyperplasia and dysplasia, the relative contribution of adipocyte size and number to the variations of fat tissue fraction is not different from controls, but the ratio of number/ size of adipocytes increased significantly in aplastic patients, suggesting that after fat cells have expanded to their maximum, a disproportional increase in number is required to fill void marrow spaces [4].

According to the Miyanishi K *et al*. the predominant lipid transport has been reported as an etiology of osteonecrosis, and a higher lipid deposition was reported in the human osteonecrosis femoral head. In bone tissue, such lipid storage is one of the major functions of marrow fat cells. This study was designed to determine whether the size of marrow fat cells differed between the corticosteroid-treated rabbits with osteonecrosis and those without osteonecrosis [5]. The marrow fat or the yellow adipose tissue (YAT) constitutes the third category of fat tissue in addition to white adipose tissue (WAT) and brown adipose tissue (BAT). The metabolic activity of YAT is not known. YAT has a moderate number of mitochondria that give its yellow appearance. YAT accumulates in areas of trabecular bone of femur, tibia and vertebrae and fills the entire marrow cavity by the third decade of human life. Marrow fat provides a localized energy reservoir for emergency situations affecting, for example, osteogenesis (eg. bone fracture, healing). YAT responds to systemic changes in energy metabolism, which is demonstrated by changes in its volume with aging. Red to yellow marrow conversion occurs to varying degrees at different skeletal sites, with a high percentage in the axial skeleton and a lower percentage in the vertebral bodies [6]. Post-mortem iliac crest has fat of 40% at 30 years which increases to 68% at 100 years. Both size and number of adipocytes increase with age. The lifetime pattern of vertebral BMF accumulation appears to differ in male and female. Before 55 years of age BMF is higher in male, but from 55-65 years, females have a steep increase in BMF while men have a gradual increase in BMF with aging. As a result, BMF is increased in females than in male. As with all fat depots, marrow fat is an endocrine organ and may directly influence the survival and function of osteoblasts and osteoclasts through the release of cytokines, adipokines and fatty acids [7]. The age-related changes in perfusion and fat content of vertebral marrow and have suggested that reduced perfusion might shift the balance between osteoblastic and osteoclastic activity, thereby altering bone density [8]. Historically, clinical measures of marrow fat required a biopsy. More recently, non-invasive measurements have become available in the research setting although not used clinically. Two techniques using magnetic resonance imaging (MRI) are widely used to assess fat in bone marrow: magnetic resonance spectroscopy (MRS) and T1-weighted MRI. Dual energy QCT can also be used. They may require relatively high levels of radiation. Single energy QCT does not give a reliable measure of marrow fat [9, 10].

Transcortical iliac crest bone biopsies were obtained from a standard location 2 cm behind and below the anterior superior iliac spine. Undecalcified sections embedded in methyl methacrylate were prepared by a standard procedure as described previously. 7-8 µm thick sections were stained in Goldner trichrome for light microscopy [11]. Histomorphometry has shown a negative association between bone marrow fat and bone formation rate. There are two types of bone and fat connection. The 'systemic connection', usually seen in obese patients, is hormonally regulated and associated with high bone mass and strength. The 'local connection' happens inside the bone marrow. Increasing amounts of bone marrow fat affect bone turnover through the inhibition of osteoblast function and survival and the promotion of osteoclast differentiation and activation. This interaction is regulated by paracrine secretion of fatty acids and adipokines [12].

## Materials and Methods:

The study protocol was reviewed and approved by the institutional ethics committee (IEC-N1/16/JUN/53/36) of Sri Ramachandra Institute of Higher education and Research, based on ICMR guidelines on biomedical research in human beings and clinical practice. The written informed consent was obtained from participants voluntarily involved in the study.

## Study participants:

Sample size: 30 bone marrow biopsy specimens received from the osteoporotic fracture patients attending for orthopaedic surgery of Sri Ramachandra Medical Centre.

## Exclusion criteria:

Patients with malignancy, stroke, hemi/paraplegia, hyperparathyroidism, thyroid disease, chronic smokers and alcoholics, cases of organ transplantation and bed ridden patients, patients on drugs like, bisphosphonates, hormone replacement therapy, vitamin-D, calcitonin and teriparatide were excluded from the study.

## BMD measurement by DEXA densitometer (GE Lunar Prodigy, Advance Bone Densitometer, US):

The BMD was determined at the neck of the femur (hip) and lumbar spine (L1-L4) by dual energy X-ray absorptiometry (DEXA) densitometer. The DEXA scan was obtained by standard procedure according to manufacturer supplied protocol for scanning and analysis. All the BMD measurements were carried out by the same well-trained technician for all the study participants. Daily quality control (QC) check was carried out by the measurement of Lunar Phantom. This every day QC check provides stable results. The BMD values were expressed as the amount of bone mineral matter per cm2 area and the obtained values. Note: - If a patient fractures in the right side neck of the femur bone, we do BMD from the left side. While fracture occurred on the left side, we measured BMD on the right side.

The T-score was determined based on WHO criteria of Normal bone mass, osteopenia osteoporosis. "Normal = T-score at or above -1.0 SD; Osteopenia = -1.0 to -2.5 SD; Osteoporosis = T- score at or below -2.5 SD"

In our study participants were grouped into two based on obtained BMD and T score of neck of femur and lumbar spine by DEXA scan. Group I - Oseopenia and Group II - Osteoporosis.

## Bone marrow fat analysis:

The bone marrow fat quantification process includes the following steps:

## Tissue fixation:

Excision bone biopsy fresh tissue specimen is immersed in the fixing solution 10 times the approximate volume of the tissue as soon as possible. The 10% formalin is used as a fixative, which penetrates at the rate of 1mm/2hours at room temperature.

## Decalcification:

Suspend the formalin fixed tissue slice placed in a cassette in the decalcifying solution. The cassette is suspended in a large quantity of decalcifying solution (more than 20 times the volume of the tissue) for the process of decalcification.

## Tissue processing:

To make tissue ready to be cut 5µm thick (4 µm thick wherever applicable)

A. Dehydration procedure: This process includes passing the tissue through a series of progressively more concentrated alcohol baths. It is done by the automatic tissue processor. Concentration of the first alcohol bath depends on the fixation and size and type of tissue. Routinely 70% alcohol is employed as the first solution.

B. Clearing: After dehydration, tissue is slightly bloated and transferred into a clearing agent if hand processing is done. Volume of the clearing agent should be 10-20 times the volume of the specimen. After the appropriate time interval, transfer the tissue from one regent to another. At least two changes should be given in a reagent like xylene, for a particular duration and then transferred to an impregnating bath.

C. Impregnation: The paraffin wax is routinely used as the impregnating and embedding media. Paraffin wax remains popular due to the ease with which a large number of tissue blocks can be processed.

In this study we used paraffin blocks (Emerck/Paxmy) of 58 - 60oC melting point were used in tissue impregnation.

## Staining:

Preparation of tissue for staining,

A. Embedding allows specimen orientation and secures the specimen in a block of wax for section cutting and storage

B. Sectioning is done on a microtome that cuts very fine section (2 - 7 µm thick)

C. This is floated out on the water bath then picked up and placed on a microscope slide.

D. The tissue on the slide is now ready for staining

The first staining step is dewaxing which uses solvent to remove the wax from the slide prior to staining. This is always done as part of the staining process. When a stain is complete the section is covered with a cover glass that makes the preparation permanent. The haematoxylin and eosin staining are used routinely.

## Histological analysis of adipose tissue and quantification of fat in marrow:

After the completion of the staining procedure, the stained specimen is placed on the microscope. The average adipocyte area, number and average diameter of fat tissue is identified for each specimen and this is enumerated under high power on 10 constructive microscopic fields.

The quantity of fat in marrow tissue was calculated using the formula = (4ϖ/3) x R1 x R2 x R3

## Statistical analysis:

The continuous variables were expressed as mean ± standard deviation and the categorical variables were presented as percentages. The correlation was found between bone marrow fat and bone mineral density measures by SPSS software, version 20 (IBM SPSS Statistics 20, US). The p value <0.05 is considered statistically significant.

## Results and Discussion:

Low bone mineral density is a potential etiologic factor for bone fractures among the patients with osteoporosis. The various risk factors causing long term decline in bone density include poor nutrition, less physical activity, increased body fat, low level of sunlight exposure [13]. The current study was planned to investigate the serum biochemical markers and bone marrow fat which is responsible for the association of low bone mineral density in patients with osteoporotic fractures. Few studies have used to measure the relationship between visceral fat and BMD by CT or MRI, in those findings visceral fat was negatively associated with bone density, content, structure, and strength [1]. In this present study result statistical significant differences with waist hip ratio in-between the group I (0.92 ± 0.02) and group II (0.98 ± 0.2), (p = 0.03). Neck of femur BMD and their T score (p < 0.0001), lumbar spine BMD and their T score (p < 0.0001) were statistically significant when compared between Group I and group II. This was represented in Table 1. Previous studies reported that higher body mass index and waist hip ratio were observed in osteopenia compared to osteoporosis.

Lipid profile disorders have been associated with low BMD in some studies. The mechanism of this relation may be directly related with the cholesterol biosynthetic pathway, which determines cholesterol levels and contributes to the activity of the osteoclast. Beneficial effect of lipid reducing drugs such as statins on BMD has been seen in most previous studies [14]. In our study results demonstrated that the higher total cholesterol and LDL cholesterol levels were observed in the osteoporosis group compared to the oseopenia group. There was a statistical significance in Total cholesterol (p < 0.001) and LDL cholesterol (p = 0.043) and we found no statistical significance of TGL and HDL cholesterol when compared with osteopenia and osteoporosis. These results may be due to minimal number of sample sizes and different study populations.

FGF21 is a member of the FGF19 subfamily; it is primarily secreted from the liver under physiological conditions and other tissues, including adipose tissue, skeletal muscle, heart and kidney. Several studies have shown that high-levels of serum FGF21 have association with the loss of bone mineral density in adults and postmenopausal women [15]. Similarly, our study populations of both the groups were identified with higher serum levels of FGF21 and a statistical significant difference (p = 0.041) was observed between Group I and Group II. The results of Table 2 represented significant negative association between neck of femur BMD versus FGF21 (R = -0.331, p < 0.01) and was a negative association of neck of femur BMD T score verses FGF21 (R = -0.443, p < 0.001). Table 3 indicates there was no significant correlation observed between FGF21 versus bone marrow fat in patients with osteoporotic fracture.

Low serum adiponectin levels to be the alarm for the compensatory mechanism of insulin resistance and low BMD. Due to its insulin-sensitive and anti-inflammatory function, adiponectin stimulates the expression of osteocalcin and differentiation of osteoblasts, and it also inhibits the accelerated adipogenesis in bone marrow, known as fatty marrow. In addition, recent observations in our study show that bone marrow fat between serum adiponetin levels may have positive, protective roles, influence and adaptive functions outside the bone tissue, due to adiponectin production, as this hormone may promote increased bone mass [16]. Based on our study report in Table 1 represented that the statistical significant difference was observed between osteopenia and osteoporosis ( p < 0.044) and in Table 3 represented that a positive significant association was observed between bone marrow fat versus serum adiponectin (R = 0.679, p < 0.001).

Bone marrow fat can indicate bone weakening nearly as well as BMD, but neither parameter alone is suitable to be used independently as an indicator. The bone marrow fat/BMD ratio showed significant diagnostic power to detect bone weakening [17]. Schellinger D *et al* reported that bone marrow fat and BMD may be inversely related. Such a relationship indicates BMD decreases and marrow fat steadily and progressively increases with age. At the cellular level, the number and size of adipocytes increase with age [18]. In our study result strengthen the above said statement (Table 3), there was statistical significant negative association between neck of femur BMD verses bone marrow fat (R = -0.618, p < 0.004) and a negative significant association between lumbar spine BMD versus bone marrow fat (R = -0.321, p < 0.01).

## Conclusion:

Aging is independent of hormonal changes and appears to contribute potentially to bone marrow adipogenesis increasing the possibility that osteoporosis is a type of lipotoxic disease. Indeed bone marrow adipocytes appear to exert a toxic effect on osteoblasts. The number of adipocytes in the marrow increases, resulting in the appearance of fatty marrow. In humans, most of the femoral cavity is occupied by fat by the third decade of life. This might induce the low bone mineral density, which leads to osteoporotic fractures.

## Financial support:

This research study was partly supported by Founder-Chancellor Shri. N.P.V Ramasamy Udayar Research Fellowship grants.

## Figures and Tables

**Table 1:** Comparison of anthropometric characteristics among the two groups

**Characteristics**	**Group I (n=12) Osteopenia Mean ± SD**	**Group II (n=18) Osteoporosis Mean ± SD**	**p value**
BMI (kg/m^2^)	26.3 ± 4.2	25.5 ± 3.7	0.084
W/H ratio	1.36 ± 0.02	0.98 ± 0.2	0.033*
NF BMD (g/cm^2^)	0.861 ± 0.03	0.645 ± 0.06	< 0.0001**
NF T score	-1.4 ± 0.4	-2.8 ± 0.43	< 0.0001**
LS BMD (g/cm^2^)	0.871 ± 0.89	0.750 ± 0.82	< 0.0001**
LS T score	-1.5 ± 0.31	-2.7 ± 0.66	<0.0001**
T.cholesterol (mg/dL)	185.5 ± 38.5	214 ± 34.7	0.001*
TGL (mg/dL)	135.8 ± 42.3	145.1 ± 42.7	0.284
HDL C (mg/dL)	46.1± 7.9	45.1 ± 7.9	0.697
LDL C (mg/dL)	113.9 ± 29.4	127.7 ± 31.3	0.043*
FGF21(pg/mL)	87.02 ± 65.2	92.3 ± 64.8	0.041*
Adiponectin (µg/mL)	3.96 ± 2.3	5.2 ± 2.9	0.044*
*Represents statistically significant
**Represents statistically highly significant

**Table 2:** Correlation between BMD and biomarkers in osteoporosis

**Group III (osteoporosis)**	**FGF21 (pg/mL)**		**Adiponectin (µg/mL)**	
	**r value**	**p value**	**r value**	**p value**
NF BMD	- 0.331*	0.01	-0.099	0.484
NF T score	- 0.443*	0.001	-0.202	0.15
LS BMD	-0.066	0.643	- 058	0.682
LS T score	0.084	0.554	-0.349*	0.01
*Represents moderate correlation
**Represents highly correlation

**Table 3:** Correlation between bone marrow fat and biomarkers and BMD in patients with osteoporosis

**Biomarkers and BMD**	**Bone marrow Fat volume (%)**	
	**r value**	**p value**
Serum adiponectin	0.679**	0.001
Serum FGF21	-0.123	0.606
Neck of Femur BMD	-0.618**	0.004
Lumbar spine BMD	-0.351*	0.01
*Represents moderate correlation
**Represents highly correlation
